# Metabolomics Profiling of Nephrotic Syndrome towards Biomarker Discovery

**DOI:** 10.3390/ijms232012614

**Published:** 2022-10-20

**Authors:** Minnie Jacob, Refat M. Nimer, Mohamad S. Alabdaljabar, Essa M. Sabi, Mysoon M. Al-Ansari, Maged Housien, Khalid M. Sumaily, Lina A. Dahabiyeh, Anas M. Abdel Rahman

**Affiliations:** 1 Metabolomics Section, Department of Clinical Genomics, Center for Genome Medicine, King Faisal Specialist Hospital and Research Centre (KFSHRC), Riyadh 11121, Saudi Arabia; 2 Department of Medical Laboratory Sciences, Jordan University of Science and Technology, Irbid 22110, Jordan; 3 Department of Internal Medicine, Mayo Clinic, Rochester, MN 55902, USA; 4 Clinical Biochemistry Unit, Pathology Department, College of Medicine, King Saud University, Riyadh 11461, Saudi Arabia; 5 Clinical Biochemistry Unit, Laboratory Medicine, King Saud University Medical City, King Saud University, Riyadh 11461, Saudi Arabia; 6 Department of Botany and Microbiology, College of Science, King Saud University, Riyadh 11451, Saudi Arabia; 7 Department of Medicine, King Faisal Specialist Hospital and Research Centre (KFSHRC), Riyadh 11211, Saudi Arabia; 8 Department of Pharmaceutical Sciences, School of Pharmacy, The University of Jordan, Amman 11972, Jordan; 9 Department of Biochemistry and Molecular Medicine, College of Medicine, Alfaisal University, Riyadh 11533, Saudi Arabia

**Keywords:** biomarker, liquid chromatography–mass spectrometry, nephrotic syndrome, metabolomics

## Abstract

Nephrotic syndrome (NS) is a kidney illness characterized by excessive proteinuria, hypoalbuminemia, edema, and hyperlipidemia, which may lead to kidney failure and necessitate renal transplantation. End-stage renal disease, cardiovascular issues, and mortality are much more common in those with NS. Therefore, the present study aimed to identify potential new biomarkers associated with the pathogenesis and diagnosis of NS. The liquid chromatography–mass spectrometry (LC–MS) metabolomics approach was applied to profile the metabolome of human serum of patients with NS. A total of 176 metabolites were significantly altered in NS compared to the control. Arginine, proline, and tryptophan metabolism; arginine, phenylalanine, tyrosine, and tryptophan biosynthesis were the most common metabolic pathways dysregulated in NS. Furthermore, alanyl-lysine and isoleucyl-threonine had the highest discrimination between NS and healthy groups. The candidate biomarkers may lead to understanding the possible metabolic alterations associated with NS and serve as potential diagnostic biomarkers.

## 1. Introduction

Nephrotic syndrome (NS) is a common urinary system disease caused by increased permeability of glomerular filtration membranes to plasma proteins resulting in a considerable loss of albumin in the urine. NS may be caused by primary (idiopathic) renal disease or other secondary causes [1]. Diabetes mellitus is the most common secondary cause, whereas focal segmental glomerulosclerosis and membranous neuropathy are the most common primary causes. NS is characterized by edema, proteinuria, hypoalbuminemia, and hyperlipidemia, which may lead to kidney failure and necessitate renal transplantation [2]. These patients are likely to have hypertension, and due to the loss of immunoglobulins in the urine, they are highly susceptible to infections such as cellulitis, peritonitis, and sepsis. NS is a kidney disease that affects children and adults; the prevalence is 16 cases per 100,000 children [3].

The mainstay treatment of NS is steroid treatment, but in the case of steroid-resistant NS patients, anticancer drugs such as vincristine have become potential alternatives. In addition, rituximab, a chimeric B-cell depleting anti–CD 20 antibody, has become a choice for patients with primary glomerular disease [4]. If children with NS are not diagnosed and treated promptly, there is a 40% chance that they may develop the condition again once they reach adulthood and ultimately progress into end-stage renal disease [5,6]. The diagnosis of NS is based on characteristic clinical symptoms, in addition to the confirmation of significant proteinuria and hypoalbuminemia [7].

Since no validated biomarkers are available for the diagnosis of NS, as well as the lack of definite and reliable treatment plans, extensive research has been performed on NS. The main challenge is enabling patients’ early diagnosis using serum samples [8,9]. Decades of research have shown that along with the development of genomics and other omics technology. Metabolomics is a promising way to identify the metabolome (global collection of small molecules typically <1500 Daltons) [10]. Additionally, metabolomics has become a promising diagnostic and prognostic tool [10]. The kidneys are notable for the degree and complexity of their intrinsic metabolic activity, performing the energy-intensive task of solute and water reabsorption from >100 L of filtrate per day [11]. Generally, metabolism in kidney diseases is particularly complex; decreased kidney function causes peripheral insulin resistance and protein energy wasting due to diabetes in most of these patients [12]. The kidneys directly impact the metabolome, including the update of many metabolites through mechanisms such as glomerular filtration, tubular secretion, catabolism, and the net release (anabolism) of several amino acids other and metabolites [13]. Changes in the metabolome, both qualitative and quantitative, have a role in the development and progression of renal disease [14]. Therefore, studying the metabolome in NS will play an important role in finding biomarkers, illuminating the path for the disease’s development mechanism, and hence contributing to early diagnosis. Currently, limited studies have been conducted to study the mechanism of NS or glomerular diseases, focusing on urinary or blood metabolomics profiling [15,16,17,18]. The growing burden of NS and its incomplete understanding of metabolic pathways is a strong challenge for blood and urine metabolomics studies on these patients. Therefore, it is urgent to find metabolomics biomarkers for NS suitable for diagnosis, follow-up, and treatment.

Recent years have seen the development of a new analytical method for biomarker identification known as chemical isotope labeling liquid chromatography–mass spectrometry (CIL LC–MS), which uses various labeling reagents to target specific functional groups based on sub-metabolomes [19,20,21]. This study aimed to explore metabolic alterations and to find new and differentially expressed biomarkers in NS patients by using CIL LC–MS targeting the amine/phenol sub-metabolomes.

## 2. Results

### 2.1. Demographics, Clinical and Molecular Features in NS Patients

The clinical and laboratory characteristics of the study cohorts are presented in Table 1. The study included serum samples collected from NS patients (n = 6) and a group of healthy controls (n = 33). NS patients’ ages ranged from 14 to 36 years, with a mean of 19.3 ± 3.44 years (SEM). The mean age of healthy control is 23 ± 1.03 years. The healthy controls were selected based on our clinical metabolomics program’s biobank, which has a broad range of samples collected from family medicine and blood bank [21]. Based on our IRB protocol, we only apply certain criteria to this sample collection, and then the algorithm provides us with the samples that match our study. The details of these clinical samples are kept hidden.

All the study participants were males and had different NS phenotypes. The mean body mass index (BMI) was 28.8 ± 4.31, and 2.5 ± 5.3 kg/m^2^, for the NS and healthy control, respectively. All NS patients had >50% secondary to minimal change disease (MCD). Mean serum creatinine and urea for the studied population were 126.8 ± 57.63 µmol/L (SEM) and 7.4 + 1.95 mmol/L (SEM), respectively. Patients had various degrees of proteinuria: dipstick proteinuria ranging from 0–3, protein random in urine 0.09–4.26 g/L, and protein/creatinine ratio ranging between 5.65–323.4 mg/mmol. Noteworthy, these laboratory tests were performed in the KFSHRC chemistry laboratory and provided by MG, where the values correspond to the time of sample acquisition for the metabolomics studies. These patients were likely in remission during sample acquisition; thus, their proteinuria is not very high or even negative [22].

### 2.2. Metabolomics Profiling for NS Compared to Control

**Figure 1:**
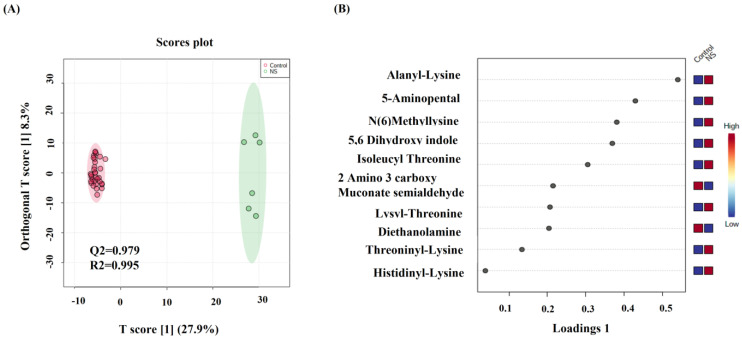
(**A**) Orthogonal partial least square discriminant analysis (OPLS-DA) score plot shows clear clustering and separation between patients with NS (n = 6) and healthy control (n = 33) representing the level of global metabolic differential expressions in NS compared to control. (**B**) The loading plot shows the regulation of metabolic expression of metabolites between NS and control.

Univariate and multivariate analyses were used to explore significantly altered metabolites in serum between NS and control. An overview of the datasets using OPLS-DA multivariate analysis revealed a clear clustering and separation between NS and control groups, reflecting significant metabolic changes in NS patients compared to healthy controls (Figure 1A). Moreover, the OPLS-DA model yielded satisfactory R2 = 0.995 and Q2 = 0.979 values (Figure 1A). To identify the most important metabolites responsible for the class separation evident in the OPLS-DA scores plot, a loading plot was generated (Figure 1B). In comparison to the control group, the NS patients exhibited higher levels of alanyl-lysine, 5-aminopentanal, and N (6)-methyl-lysine as shown by the loading plot in Figure 1B. Whereas 2-amino-3-carboxymuconate semialdehyde and diethanolamine were decreased in NS compared with the control group.

**Figure 2:**
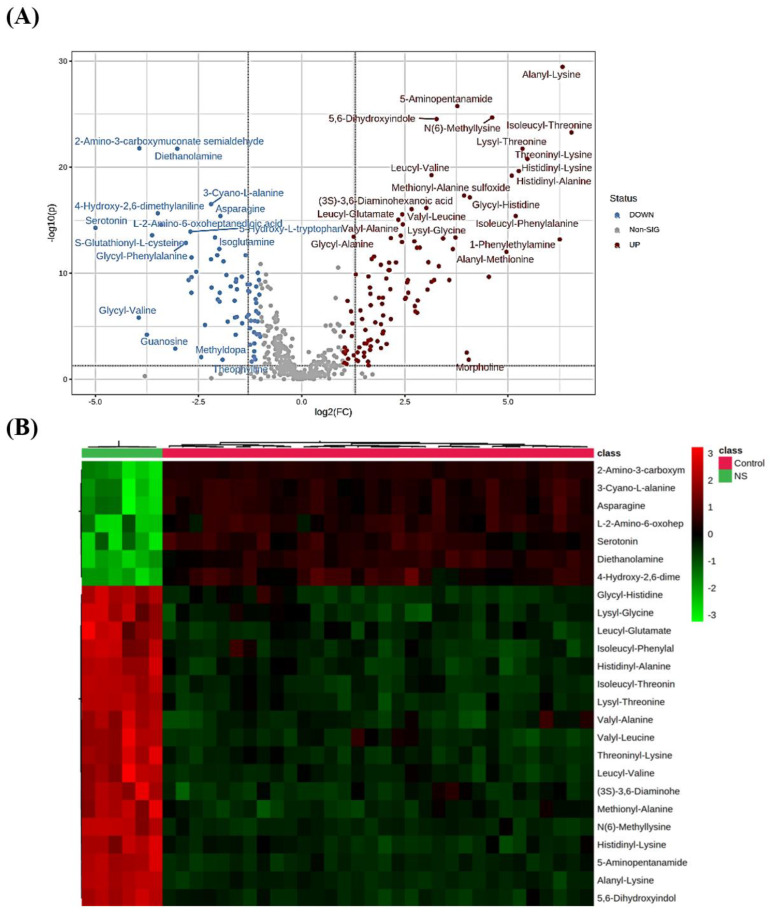
Binary comparison for serum samples of patients with NS and healthy controls. (**A**) Dysregulated metabolites between NS vs. control with fold change cut-off of 2 and false discovery rate (FDR) threshold of 0.05; up-regulated = 105 (red), down-regulated = 71 (blue). (**B**) Hierarchal clustering (HAC) and heat map analysis of the top 25 significantly altered metabolites between the two study groups; controls (red), and NS (green).

Univariate analysis using a volcano plot was performed by applying a false discovery rate (FDR) and fold change (FC) thresholds of 0.05 and 2, respectively, to facilitate the screening of differential metabolites. As shown in Figure 2A, 105 and 71 metabolites were significantly up- and down-regulated, respectively. Similar to multivariate results, alanyl-lysine had the most significant increase in its level in NF compared to controls. Among the significantly increased metabolites in NS were 5-aminopentanal, 5,6-dihydroxyindole, and N6-methyl-lysine, while 2-aminopyridine-3-carbaldehyde and diethanolamine were among the significantly decreased metabolites in NS compared to control (Figure 2A).

A heat map visualization of the top 25 metabolites altered between NS and control groups is shown in Figure 2B. As noted, serotonin, asparagine, and diethanolamine were significantly decreased in NS compared to healthy controls. On the other hand, the level of lysyl-glycine, alanyl-lysine, and glycyl-alanine were significantly higher in NS.

**Figure 3:**
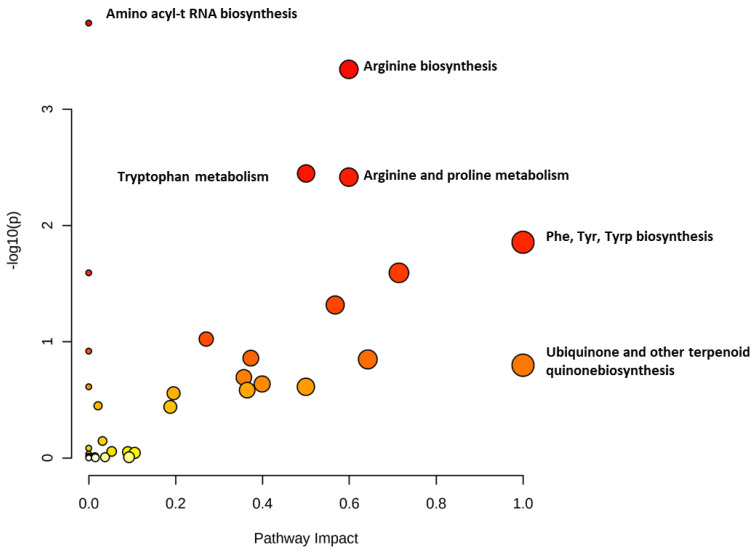
Pathway analysis shows the altered pathways in NS including amino acid metabolism and ubiquinone biosynthesis. The node color and size of the circle reflect the *p*-value and the pathway impact value, respectively.

Significantly altered metabolites were subjected to pathway analysis to identify significantly dysregulated pathways in NS. The pathway analysis identified amino acyl-t RNA and arginine biosynthesis pathways to be the most perturbed in NF compared to control cohorts, followed by different amino acid metabolism pathways, including arginine, tryptophan, and proline (Figure 3). These changes are represented by the circles (reflecting pathway impact) and size (statistical significance, *p*-value) in Figure 3.

**Figure 4:**
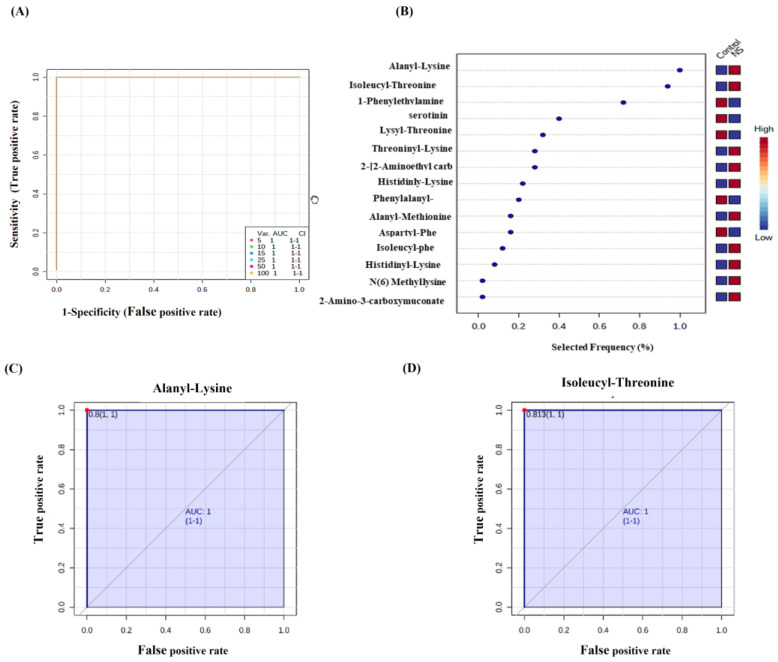
Receiver operating characteristics (ROC) curve and loading Plots for significant metabolites in serum of patients with NS. (**A**) ROC was generated using the PLS-DA model showing area under the curve (AUC) for the top five variants = 1. (**B**) Frequency percentage plot of the altered metabolites in NS patients when compared with controls. (**C**) Alanyl-Lysine (AUC = 1) and (**D**) Isoleucyl-Threonine (AUC = 1) were up-regulated in NS patients when compared with control.

Receiver operating characteristic (ROC) analysis was performed to identify metabolites that have the potential to act as potential biomarkers and to evaluate the diagnostic accuracy of these metabolites. PLS-DA was used as a classification and feature ranking approach to creating a multivariate exploratory ROC analysis. The top metabolites that displayed high classification ability were alanyl-lysine, isoleucyl-threonine, 1-phenylethylamine, serotonin, lysyl-threonine, and threonine-lysine (Figure 4B). Figure 4A shows that the top five metabolites in the exploratory ROC curve had an area under the curve (AUC) of 1. Two metabolites, alanyl-lysine, and isoleucyl-threonine, were up-regulated in NS compared to control with an AUC of 1 (Figure 4C) and (Figure 4D), respectively, showing the highest discriminatory power.

Data were normalized, transformed, and scaled by median log and Pareto scaling to make sure all the data were under Gaussian distribution.

## 3. Discussion

NS has emerged as one of the most prevalent primary kidney disorders, and its progressive forms are associated with an increased risk of developing chronic renal disease (CKD) [23]. Additionally, NS is a diverse condition that may have a variety of underlying causes; making a definitive diagnosis is often inaccurate, particularly in primary care settings [8,24]. Therefore, it is necessary to identify novel biomarkers to aid in early diagnosis and facilitate treatment and disease follow-up.

Despite the growing field of metabolomics as a promising technique to study disease for diagnostic and therapeutic purposes by measuring the overall expression of small molecules (<1500 Da) [25], few studies have focused on NS in humans [1,9]. We collected random samples, although there could be a very marginal effect of fasting on the metabolomics profile, this effect is not major and was normalized with the fold change filter [26]. Therefore, CIL LC–MS robust method for metabolomics biomarker discovery was applied, to identify the predictive biomarkers for NS based on the serum metabolite changes allowing the detection of very low abundant (pmol to fmol) metabolites and ^13^C-labeled pool served as an internal standard and compensated for the fluctuations in MS response.

3C-labeled metabolite and its corresponding 12C-labeled metabolite in a 12C-/13C-mixture were detected as peak pairs and their concentration was found based on the peak area ratio and these values were used to quantify the individual metabolite in the samples. Mass and retention time was matched with the labeled standard library for positive metabolite identification. Putative identification was achieved by matching the mass to the metabolites in HMDB, the mass accuracy tolerance window was set at 10 ppm [27].

In the present work, metabolomics analysis of NS has detected an alteration in the amino acids’ metabolic pathways including arginine, proline, and tryptophan metabolism; arginine, phenylalanine, tyrosine, and tryptophan biosynthesis. These results are consistent with the well-known dysregulation of protein anabolism and catabolism in NS patients [28,29]. Moreover, inadequate protein intake and amino acid depletion are associated with NS [28]. The metabolites of amino acids are linked with the pathogenesis of NS [30,31]. Metabolites of tryptophan, such as 3-indoxyl sulfate, are associated with a substantial decrease in eGFR [31]. Tryptophan is converted to niacin and quinolone through the kynurenine pathway, and this pathway is activated in renal insufficiency and CKD [32]. Furthermore, 2-amino-3-carboxymuconate semialdehyde is an intermediate in the metabolism of tryptophan in the tryptophan-niacin catabolic pathway is decreased in renal diseases as reported in our study [21]. Consistent with our findings, Zhang et al. reported that arginine and proline metabolism were among the metabolic pathways perturbed in primary nephrotic syndrome (PNS) patients [33]. In addition, PNS had decreased tryptophan and increased arginine level [33]. Shouman et al. reported that nitric oxide (NO) synthesized in the body from arginine by the enzyme nitric oxide synthase (NOS) might be involved in the pathogenesis of NS and its complications [30].

One of the metabolic processes that is dramatically altered between NS and healthy control is the aminoacyl-tRNA biosynthesis pathway. During translation, which is the most important step of protein formation, aminoacyl-tRNA plays an essential role as substrates bind that bind to an amino acid by an aminoacyl-tRNA synthetase. The alteration in aminoacyl-tRNA in our study is consistent with previous findings related to kidney diseases [21,34].

The N6-methyllysine level was significantly higher in NS than in control herein. The L-lysine derivative known as N6-methyl-L-lysine has a methyl group replacing one of the hydrogens on the N6 carbon atom that occur in certain muscle proteins [35,36]. The elevation of L-lysine derivative in the blood of patients with NS is associated with a hypercatabolic state expressed as an exacerbated degradation of muscle mass [37]. Moreover, our result showed an elevated level of 5-aminopentanamide in patients with NS, a product of the decarboxylation of lysine [38]. Our research is the first to report the increase in 5-aminopentanamide level in NS which may be linked to changes in kidney function and the hypercatabolic state in NS [39,40].

The metabolite 5,6-dihydroxyindole significantly increased in the NS group, is the first product of dopamine oxidation. Dopamine oxidation has been reported to play a role in the decline of proximal tubular mitochondrial and lysosomal function as well as in renal inflammation [41]. The previous might explain the higher level of 5,6-dihydroxyindole in NS.

In our study, alanyl-lysine, and isoleucyl-threonine had the highest discrimination ability between NS and the control group. These metabolites are dipeptides composed of two amino acids joined by peptide bonds resulted from an incomplete breakdown product of protein digestion or protein catabolism [42]. These dipeptides are considered secondary metabolites which are not directly involved in primary metabolic processes but arise from the incomplete metabolism of the secondary metabolites [43]. Patients with NS often have a predominant state in their bodies that is characterized by faster catabolism, increased protein intake, and loss, as well as malnutrition [44]. As the illness advances, these symptoms are accompanied by inflammatory hypoproteinemia and hyperlipidemia [45]. In addition, patients with kidney diseases have higher plasma levels of numerous products derived from amino acids and proteins, which are principally caused by reduced urine clearance by the kidney [46]. To the best of our knowledge, the dipeptide isoleucyl-threonine has been identified in human biofluids for the first time, which can serve as a potential diagnostic biomarker for NS.

This study has some limitations, for instance, the small sample size and the absence of a separate external validation cohort. However, our study is one of the few papers identifying several specific metabolite differences between NS and healthy control. Certainly, our results represent the starting point for more in-depth studies.

## 4. Materials and Methods

### 4.1. Chemicals

The LC–MS grade reagents, including water, acetonitrile (ACN), methanol, and formic acid, were purchased from Fisher Scientific (Ottawa, ON, Canada), and 13C-dansyl chloride was available from the University of Alberta (http://mcid.chem.ualberta.ca (accessed on 19 January 2020)) as described earlier [47].

### 4.2. Characteristics of the Study Population

Blood samples were collected in plain tubes (Vacutainer, BD Biosciences, San Jose, CA, USA) from 6 patients with clinically confirmed NS from the Renal clinics at King Faisal Specialist Hospital and Research center (KFSHRC) from 2018–2020. The blood samples were centrifuged to obtain serum and stored at −80 °C for metabolomics analysis. Our Inclusion criteria included healthy controls (n = 33) who visited the clinic for routine clinical care and were free of any kidney diseases were recruited to participate in this study, along with the above mentioned 6 confirmed NS patients. A baseline questionnaire was also collected, including the clinical symptoms, laboratory findings, (creatinine, urea, and proteinuria levels), allergies, and family history. Patients who were enrolled in other clinical studies, those who were unwilling to provide informed consent, and whose sample amount was insufficient were excluded from the arm of this study. The protocol was revised and approved by the Research Ethics committee at KFSHRC (project #2160027).

### 4.3. CIL LC–MS Metabolomics Profiling

Serum samples were labeled by 12C dansyl-chloride (DnsCl), while a pooled sample was generated by mixing all individual samples and then labeled by 13C-DnsCl to serve as a reference for all the 12C-labeled individual samples, as described earlier [19].

Each sample was normalized before LC–MS analysis. LC–UV quantitation was performed to determine the total concentration of dansyl-labeled metabolites. Before injection onto LC–MS, each 12C-labeled sample was mixed with the same molar amount of the 13C-labeled pooled sample. All labeled metabolites were identified as peak pairs on mass spectra, and the peak area ratios were used for quantitative metabolomics analysis. Thermo Fisher Scientific Dionex Ultimate 3000 UHPLC System (Sunnyvale, CA, USA) linked to a Bruker Maxis II quadrupole, time-of-flight (Q-TOF) mass spectrometer (Bruker, Billerica, MA, USA) was used to analyze the serum samples. The LC column was an Agilent reversed-phase Eclipse Plus C18 column (2.1 mm × 10 cm, 1.8 μm particle size, 95 Å pore size), while the mobile phase A was 0.1% (*v*/*v*) formic acid in 5% (*v*/*v*) ACN, and solvent B was 0.1% (*v*/*v*) formic acid in acetonitrile. The LC gradient was as follows: t = 0 min, 20% B; t = 3.5 min, 35% B; t = 18 min, 65% B; t = 21 min, 99% B; t = 34 min, 99% B, with a flow rate of 0.18 mL/min. The MS conditions were as follows: polarity, positive; dry temperature, 230 °C; dry gas, 8 L/min; capillary voltage, 4500 V; nebulizer, 1.0 bar; endplate offset, 500 V; spectra rate, 1.0 Hz.

### 4.4. Data Processing and Statistical Analysis

Bruker Daltonics Data Analysis 4.3 software was used for metabolomics profiling. IsoMs extracted the peak pairs after filtering out the redundant peak pairs, and data were aligned based on accurate mass and retention times [48]. A univariate analysis was performed to identify the significantly differentially expressed metabolites (fold-change of greater than 1.5 or less than 0.67 with q-value (false discovery rate) less than 0.05). The principal component analysis (PCA) and partial least squares discriminant analysis (PLS-DA) plots were performed using Iso MS Pro. (NovaMT Inc.). The metabolites were positively identified by searching against DnsID Library (www.mycompoundid.org) using retention time and accurate mass [49]. MyCompoundID library, which contains 8021 known human metabolites and 375,809 predicted metabolites (www.mycompoundid.org (19 January 2020)) was used for putative identification [50].

The raw data from the metabolomics experiments were statistically analyzed using MetaboAnalyst software version 5.0 (McGill University, Montreal, QC, Canada). Data were normalized, and the total sample median was used for normal distribution evaluation. Log transformation was used to adjust the sample differences, and Pareto scaling was performed for a comparative review of individual features. An FDR-corrected *p*-value < 0.05 was used and the values were reported as mean ± SEM. Supervised multiple regression analysis was performed to discriminate between two different datasets, and orthogonal partial least squares projection to latent structure discriminant analysis (OPLS-DA) was generated as part of the chemometric analysis [51]. The receiver operating characteristic (ROC) curves were constructed using the PLS-DA method for global analysis.

## 5. Conclusions

We created an untargeted metabolomics profile for patients with NS by using a chemical isotope-labeled mass spectrometry-based metabolomics technique. Metabolomic analysis between NS and control groups identified several dysregulated metabolites (105 and 71 were significantly up- and down-regulated, respectively, in NS compared to control). The altered metabolites related to arginine, proline, and tryptophan metabolism, arginine, phenylalanine, tyrosine, and tryptophan biosynthesis. Two metabolites, alanyl-lysine, and isoleucyl-threonine had the highest discrimination between NS and the healthy group. Moreover, our results showed novel potential biomarkers for NS, such as 5-aminopentanamide and isoleucyl-threonine. However, these biomarker panels have to be validated in a larger, multicenter trial before they can be used in clinical practice.

## Figures and Tables

**Table 1:** Clinical characteristics of NS patients.

Patient	NS Phenotype/ Renal Disease	Age (Yrs)	BMI	Body Weight (Kg)	SBP (mmHg)	DBP (mmHg)	eGFR (>60 mL/min/ 1.73 m^2^)	Serum Creatinine (64–115 μmol/L)	Urea (2.3–6.7 mmol/L)	Protein Dipstick	Protein Random Urine (g/L)	Pr/Cr Random Urine Ratio (mg/mmol)
NS-1	Congenital	15	14.6	29.5	138	87	N/A	412	12.8	3+	1.52	323.4
NS-2	MCD, Relapsing	36	29.1	72	113	75	>60	65	4.4	trace	0	5.65
NS-3	FSGS, Steroid-dependent	19	17.6	45	131	91	>60	57	4.6	2+	0.49	32.77
NS-4	Steroid-dependent, FSGS + MCD	18	40.4	106	128	75	>60	87	14.2	3+	4.26	132.3
NS-5	Steriod-dependent, MCD	14	37	83.3	132	74	N/A	42	3.3	Negative	0.17	10.69
NS-6	Steriod-dependent, MCD	14	34	72.5	108	62	N/A	98	5.2	2+	0.69	127.78
Average ± SEM		19.3 ± 3.44	28.8 ± 4.31	68.1 ± 11.14	125 ± 4.82	77.3 ± 4.23		126.8 ± 57.63	7.4 ± 1.95		1.2 ± 0.65	105.4 ± 49.31

Abbreviations—MCD: minimal change disease, FSGS: focal segmental glomerulosclerosis, BMI: body mass index, SBP: systolic blood pressure, DBP: diastolic blood pressure, eGFR: estimated glomerular filtration rate. Data are presented as mean ± SEM.

## Data Availability

Metabolomics data were deposited to the EMBL-EBI MetaboLights database with the identifier MTBLS6183. The complete dataset can be accessed at https://www.ebi.ac.uk/metabolights/ MTBLS6183 (accessed on 5 October 2022).
